# The role of aldosterone blockade in murine lupus nephritis

**DOI:** 10.1186/ar2353

**Published:** 2008-01-15

**Authors:** Seetha U Monrad, Paul D Killen, Marc R Anderson, Amanda Bradke, Mariana J Kaplan

**Affiliations:** 1 Division of Rheumatology, Internal Medicine, University of Michigan Medical School,, Ann Arbor, Michigan 48109, USA; 2 Department of Pathology, University of Michigan Medical School,, Ann Arbor, Michigan 48109, USA

## Abstract

**Background:**

The purpose of this study was to examine the effect of aldosterone receptor blockade on the immunopathogenesis and progression of nephritis in the (NZB × NZW) F_1 _murine lupus model.

**Methods:**

Female NZB/W F_1 _mice (11 weeks old) were treated daily with 25 or 50 mg/kg oral spironolactone or vehicle. Proteinuria, renal function, and serum autoantibody levels were monitored. Renal histopathology, immune complex deposition, and immunohistochemistry were analyzed at various time points. Targeted microarray analysis was performed on renal tissue, with subsequent real-time PCR analysis of several differentially expressed genes.

**Results:**

Treatment with spironolactone was well tolerated by the mice throughout the course of their disease progression, with no significant differences in azotemia or serum potassium levels between vehicle-treated and spironolactone-treated animals. By 36 weeks of age, fewer spironolactone-treated mice developed nephrotic range proteinuria as compared with the control mice (control 70.8%, 25 mg/kg spironolactone 51.3%, and 50 mg/kg spironolactone 48.6%). Compared with control mice, mice treated with 25 mg/kg spironolactone had significantly lower serum anti-single-stranded DNA levels (2,042 μg/ml versus 1,036 μg/ml; *P *= 0.03) and anti-double-stranded DNA levels (3,433 μg/ml versus 614 μg/ml; *P *= 0.05). Spironolactone-treated mice exhibited decreased histopathologic evidence of inflammation and tissue damage, as compared with control mice. Additionally, spironolactone treatment resulted in decreased expression in the kidney of several inflammatory and proapoptotic genes, including those encoding interferon-γ, B lymphocyte stimulator (BlyS), tumor necrosis factor related apoptosis inducing ligand (TRAIL), tumor necrosis factor related weak inducer of apoptosis (TWEAK), and Fas ligand.

**Conclusion:**

Aldosterone receptor blockade is safe and well tolerated in progressive murine lupus nephritis, and it results in decreased levels of clinical proteinuria, lower serum levels of autoantibodies, and decreased kidney damage. It appears to modulate inflammatory changes during the progression of glomerulonephritis and may also have a previously undescribed role in attenuating apoptosis.

## Introduction

Renal involvement is a major cause of morbidity and mortality in systemic lupus erythematosus (SLE). The underlying pathogenesis of the diverse clinical and histopathologic manifestations of lupus nephritis is still not well understood, although a complex interplay of genetic factors, autoantibodies (autoAbs), inflammatory responses, and aberrant apoptosis has been implicated [1]. Lupus nephritis is often referred to as the prototypic immune complex (IC) disease, in which glomerular deposition of circulating ICs or *in situ *formation of renal autoantigen-autoAb complexes results in the recruitment of inflammatory cells, cytokine and vasoactive substance release, and complement activation [2]. In addition to inflammatory nephritis, lupus renal involvement can also manifest as a fibrotic, atrophic nephropathy with significant renal functional impairment and potential progression to end-stage disease. Although this manifestation can be the progressive result of earlier unchecked inflammation, this may not always be the case, and the precise relationship between acute inflammatory and chronic fibrotic nephropathy is unclear [3-5]. Thus, therapeutics focusing solely on suppression of inflammation may be inadequate in preventing terminal fibrotic damage.

The renin-angiotensin-aldosterone axis, as a major homeostatic regulator of renal function, has long been known to participate in the pathogenesis of renal disease [6,7], although the specific role played by aldosterone in chronic renal disease has only recently received attention [8,9]. In addition to the hemodynamic effects of sodium retention and systemic vasoconstriction, aldosterone has a number of well documented profibrotic effects. It can promote fibrosis in target organs via direct effects on vascular smooth muscle cells, endothelial cells, renal fibroblasts, and mesangial cells; additionally, aldosterone modulates expression of various profibrotic mediators, including transforming growth factor-β_1_, plasminogen activator inhibitor-1, and reactive oxygen species (for reviews [10-12]). Aldosterone also exerts proinflammatory effects in the kidney and other tissues [13,14], such as leukocyte infiltration and increased expression of inflammatory cytokines. In addition, aldosterone can generate cytosolic cation imbalances in mononuclear cells, resulting in an immunostimulatory phenotype [15]. These findings suggest an intriguing potential immunomodulatory role for aldosterone, which could be important in the pathogenesis and progression of lupus nephritis.

A number of diverse animal models of renal dysfunction have demonstrated that aldosterone blockade attenuates proteinuria and histopathologic parameters of renal injury [12,16-22]. Additionally, overactivity of the renin-angiotensin axis and the beneficial effects of angiotensin blockade in lupus nephritis has been demonstrated [23,24]. However, the role of aldosterone and the effects of aldosterone blockade on lupus nephritis specifically have not been characterized. We examined the effect of the aldosterone receptor antagonist spironolactone on the development and progression of nephritis in the NZB/W F_1 _murine model of SLE.

## Materials and methods

### Animals

Female NZB/W F_1 _mice, aged 6 to 8 weeks, were purchased from the Jackson Laboratory (Bar Harbor, ME, USA) and housed in individual cages in a specific pathogen-free barrier facility at the University of Michigan. All experiments were approved by the institutional committee for animal use.

### Reagents

Spironolactone (Sigma, St. Louis, MO, USA) was suspended in a vehicle of 1:1 mixture of Ora-Plus (Paddock Laboratories, Minneapolis, MN, USA) and strawberry syrup, purchased from the University of Michigan Hospital Stores (Ann Arbor, MI, USA). Sugar-free gelatin (Jell-O brand) was purchased commercially.

### Spironolactone treatment and monitoring

Preliminary experiments revealed spironolactone to be insoluble at our desired concentrations in nontoxic solvents. Therefore, spironolactone suspension or vehicle alone was mixed with gelatin at varying concentrations contained in a 1 cm^3 ^cube of gelatin; gelatin/drug cubes were made on a daily basis. The mice routinely ate the entire amount provided.

Eleven-week-old prenephritic mice were fed daily either spironolactone 50 mg/kg (*n *= 16), spironolactone 25 mg/kg (*n *= 16), or vehicle alone (*n *= 16; referred to as 'control'). The doses chosen were based on those safely tolerated in other murine nephropathy models in the literature [19,25] and are 10-fold to 20-fold higher than those routinely received by humans (about 2.5 mg/kg per day). Randomly sampled mice from each group were periodically weighed and tail cuff pressures obtained. To monitor for hyperkalemia and uremia, mice were bled via retro-orbital approach every 4 weeks; serum was immediately isolated using serum separator tubes and then frozen at -80°C until use. Serum autoAbs were measured at baseline and at ages 25 and 36 weeks. Proteinuria was determined by collecting spot urine samples, initially every 4 weeks and then weekly after age 25 weeks, and analyzed immediately upon collection. At 25 weeks old, half of the mice in each group were killed, whereas the remaining mice continued their respective treatments and blood and urine collection until 36 weeks, at which time they were killed. Additionally, five untreated 11 week old mice were killed at the beginning of the protocol to determine baseline parameters.

### Serum and urine measurements

Proteinuria was measured using Uristix (Bayer, Elkhart, IN, USA) and expressed semiquantitatively: 0 (none-trace), 1+ (30 mg/dl), 2+ (100 mg/dl), 3+ (300 mg/dl), 4+ (>2000 mg/dl). Serum potassium and blood urea nitrogen levels were assayed using colorimetric kits (Stanbio Laboratory, Boerne, TX, USA), as per the manufacturer's protocols. Levels of autoAbs against single-stranded DNA (ssDNA) and double-stranded DNA (dsDNA) were determined by enzyme-linked immunosorbent assay (Alpha Diagnostic, San Antonio, TX, USA), as per the manufacturer's protocols. The detection limits were 400 ng/ml and 156 ng/ml for anti-dsDNA and anti-ssDNA antibodies, respectively.

### Kidney preparation for immunohistochemistry, immunofluorescence, and RNA extraction

Mice were fully anesthetized with pentobarbital. The abdomen was dissected and the abdominal aorta, inferior vena cava, kidneys, and renal vasculature were exposed. The aorta was cannulated with an angiocatheter, the inferior vena cava punctured, and the vasculature perfused with ice cold phosphate-buffered saline (Invitrogen, Carlsbad, CA, USA) at a rate of approximately 12 ml/min for 5 minutes. The left renal vascular bundle was ligated with surgical suture and the kidney removed. A section of cortex was snap frozen in liquid nitrogen and stored at -80°C for immunofluorescence staining; the remaining tissue was homogenized in TriPure isolation reagent (Roche, Indianapolis, IN) and frozen for future RNA extraction.

The remaining right kidney was fixed by flushing the vasculature with ice cold 4% paraformaldehyde for 10 minutes, removed, and placed in 10% formalin for future paraffin embedding and sectioning.

### Histopathology studies

Kidneys fixed in formalin were processed, paraffin embedded, and sectioned at 2 to 3 μm thickness. Hematoxylin and eosin or Masson's trichrome stained sections were examined and graded by one of the authors (PDK) in a blinded manner. A semiquantitative scoring system (0 = no involvement, 1 = minimal involvement [0% to 25% of section], 2 = moderate involvement [26% to 50% of section], and 3 = severe involvement [>50% of section]) was used to assess 13 different parameters of activity and chronicity (mesangial hypercellularity, mesangial deposits, mesangial sclerosis, endocapillary cellular infiltrate, subepithelial and subendothelial deposits, capillary thrombi, capillary sclerosis, cellular or organized crescents, synechiae, tubular atrophy, and interstitial fibrosis). An activity index and a chronicity index were generated by compiling scores from groups of related parameters (for activity: mesangial hypercellularity, mesangial deposits, and endocapillary cellular infiltrate; for chronicity: interstitial fibrosis, tubular atrophy, synechiae, organized crescents, and capillary sclerosis).

### Immunohistochemistry/immunofluorescence staining of kidney sections

Immunohistochemistry for rat anti-mouse F4/80 (Serotec, Raleigh, NC, USA) or concentration-matched rat IgG (Southern Biotech, Birmingham, AL, USA) was performed on paraffin sections using avidin-biotin complex techniques (Vectastain Elite ABC kit, Vector Laboratories, Burlingame, CA, USA) and visualization with diaminobenzidine. Scoring was performed in a blinded manner, as previously described [26]. Immunofluorescence staining was performed as described previously [27] using FITC goat anti-mouse C3 (Cappel Laboratories, Malvern, PA, USA) and Alexa Fluor 594 F(ab')_2 _goat anti-mouse IgG (Invitrogen). Glomerular staining was graded according to intensity on a 0 to 4+ scale (0 = no staining; 4+ = maximum intensity staining).

### RNA analysis

Renal RNA was extracted using TriPure as per the manufacturer's protocol and further purified using an RNEasy Mini Kit (Qiagen Inc., Valencia, CA, USA). RNA was pooled from mice within the same age and treatment group. Targeted microarrays (Superarray, Frederick, MD, USA) looking at inflammatory cytokine (MM-003) and extracellular matrix-related genes (OMM-013) were performed as per the manufacturer's protocol; gene expression relative to age-matched, vehicle-treated control animals was determined using the manufacturer's software, corrected for background and normalized to the median. The data discussed in this report have been deposited in National Center for Biotechnology Information's Gene Expression Omnibus (GEO) [28] and are accessible through GEO Series accession number GSE10144. Pooled RNA was frozen at -80°C before use in real-time PCR.

### Real-time PCR

cDNA was generated using Superscript 2 (Invitrogen), reverse transcriptase, and random hexamers. The primers used are summarized in Table 1. Real-time PCR was carried out in triplicate on an Applied Biosystems 7900 HT sequence detection system using SYBR green master mix (Applied Biosystems, Foster City, CA, USA), optimized concentrations of forward and reverse primers, and 25 ng cDNA. PCR conditions were as follows: one cycle of 95°C for 4 minutes, then 40 cycles of 95°C for 15 seconds and 60°C for 1 minute. Fold induction/repression was determined using the 2^-ΔΔCt ^relative quantification method, normalized against GAPDH (glyceraldehyde 3-phosphate dehydrogenase).

**Table 1:** Primers used

Gene	Primer
IFN-γ	Forward: 5'-CAT TGA AAG CCT AGA AAG TCT GAA TAA C-3'
	Reverse: 5'-TGG CTC TGC AGG ATT TTC ATG-3'
BlyS	Forward: 5'-CAG GAA CAG ACG CGC GAT TTC-3'
	Reverse: 5'-GTT GAG AAT GGC GGC ATC C-3'
TRAIL	Forward: 5'-GAT CAC TCG GAG AAG CAA CTC A-3'
	Reverse: 5'-GAG AGG ACT CCC AGG ATT CAA TG-3'
TWEAK	Forward: 5'-CGA GCT ATT GCA GCC CAT TAT-3'
	Reverse: 5'-ACC TGC TTG TGC TCC ATC CT-3'
FasL	Forward: 5'-GCA CAT AGC CAA CCC CAG TAC AC-3'
	Reverse: 5'-GCC ACC TTT ATT ATA CTT CAC TCC AG-3'

BlyS, B lymphocyte stimulator; FasL, Fas ligand; IFN, interferon; TRAIL, tumor necrosis factor-related apoptosis-inducing ligand; TWEAK, tumor necrosis factor-like weak inducer of apoptosis.

### Statistics

Kaplan-Meier plots for survival and proteinuria were compared using nonparametric log-rank testing. Serum chemistry and autoantibody levels were compared using Student's *t*-test. Renal histopathology scores were analyzed using nonparametric Kruskall-Wallis testing.

## Results

### Clinical effects of spironolactone treatment

There were no significant differences between mice in different treatment groups with regard to survival, weight, blood pressure, serum urea nitrogen, or serum potassium levels at various time points throughout the study (data not shown).

### Effects of spironolactone on proteinuria

**Figure 1:**
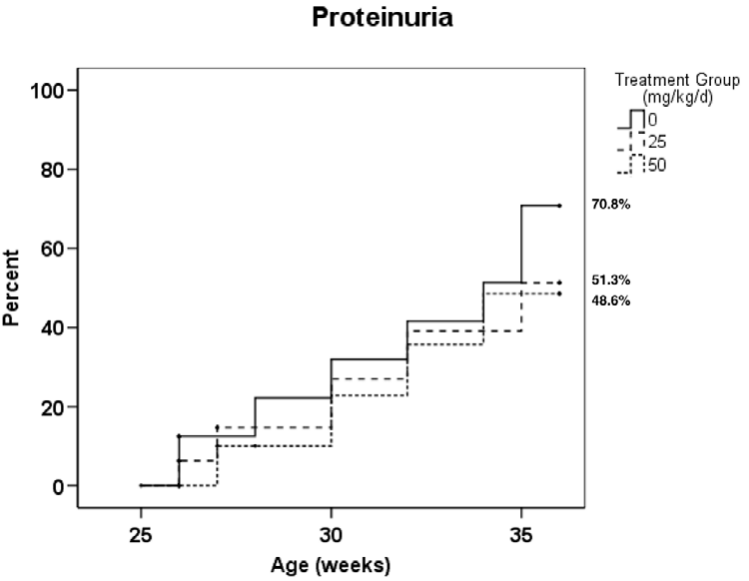
Effect of spironolactone on the development of nephrotic range proteinuria. The age at onset of nephrotic range proteinuria was compared between treatment groups. At the 36-week time point, 70.8% of the control mice had nephrotic proteinuria (as opposed to 51.3% and 48.6% of the mice treated with 25 mg/kg per day and 50 mg/kg per day, respectively). Sixteen animals were included in each group at the start of the study. *P *= 0.331, control versus spironolactone 50 mg/kg per day.

Mice were tested initially on a biweekly and then weekly basis for proteinuria. In general, mice began to exhibit sustained low-grade proteinuria at around 20 weeks of age. We determined the time to onset of nephrotic range proteinuria, defined as a spot urine analysis of 3+ (300 mg/dl) or greater, sustained for 2 weeks or more. Although there were no statistically significant differences between the three groups over the entire course of the study in development of nephrotic range proteinuria, by age 36 weeks fewer spironolactone-treated mice had developed nephrotic range proteinuria compared with the control mice (control 70.8%, 25 mg/kg spironolactone 51.3%, 50 mg/kg spironolactone 48.6%; *P *= 0.331 comparing control with spironolactone 50 mg/kg; Figure 1).

### Effects of spironolactone on serum autoantibodies

**Figure 2:**
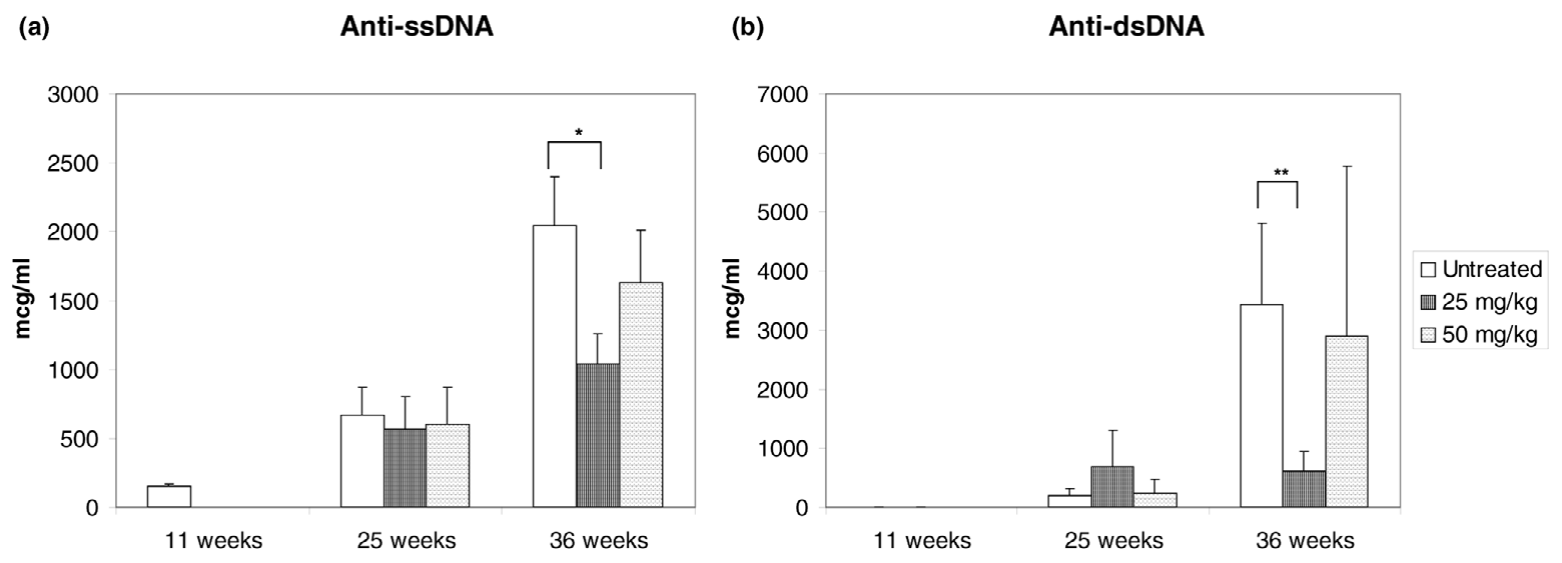
Effect of spironolactone on autoantibody levels. At 36 weeks of age, there were significant differences in **(a) **anti-single-stranded DNA (ssDNA) and **(b) **anti-double-stranded DNA (dsDNA) antibody levels between control mice and mice treated with 25 mg/kg per day spironolactone. Results represent the mean ± standard error in six to eight mice/group. *P = 0.02, **P = 0.05.

Serum levels of autoAbs were determined at baseline, age 25 weeks, and age 36 weeks from randomly sampled mice in each treatment group. There were significant differences in anti-ssDNA antibody levels (Figure 2a) between control mice and mice treated with 25 mg/kg per day spironolactone by 36 weeks of age (mean level: control 2,042 ± 356 μg/ml, treated 1,036 ± 225 μg/ml; *P *= 0.02), whereas no significant differences between control mice and mice treated with 50 mg/kg per day spironolactone (mean: 1,628 ± 381 μg/ml; *P *= 0.40) were observed. Additionally, there were significant differences in anti-dsDNA antibody levels (Figure 2b) between the control and 25 mg/kg per day spironolactone groups (mean: control 3,433 ± 1,370 μg/ml, treated 614 ± 336 μg/ml; *P *= 0.05) but not the 50 mg/kg per day spironolactone group (mean 2,890 ± 1,569 μg/ml; *P *= 0.78).

### Effects of spironolactone on renal histopathology, immune complex deposition, and macrophage infiltration

**Figure 3:**
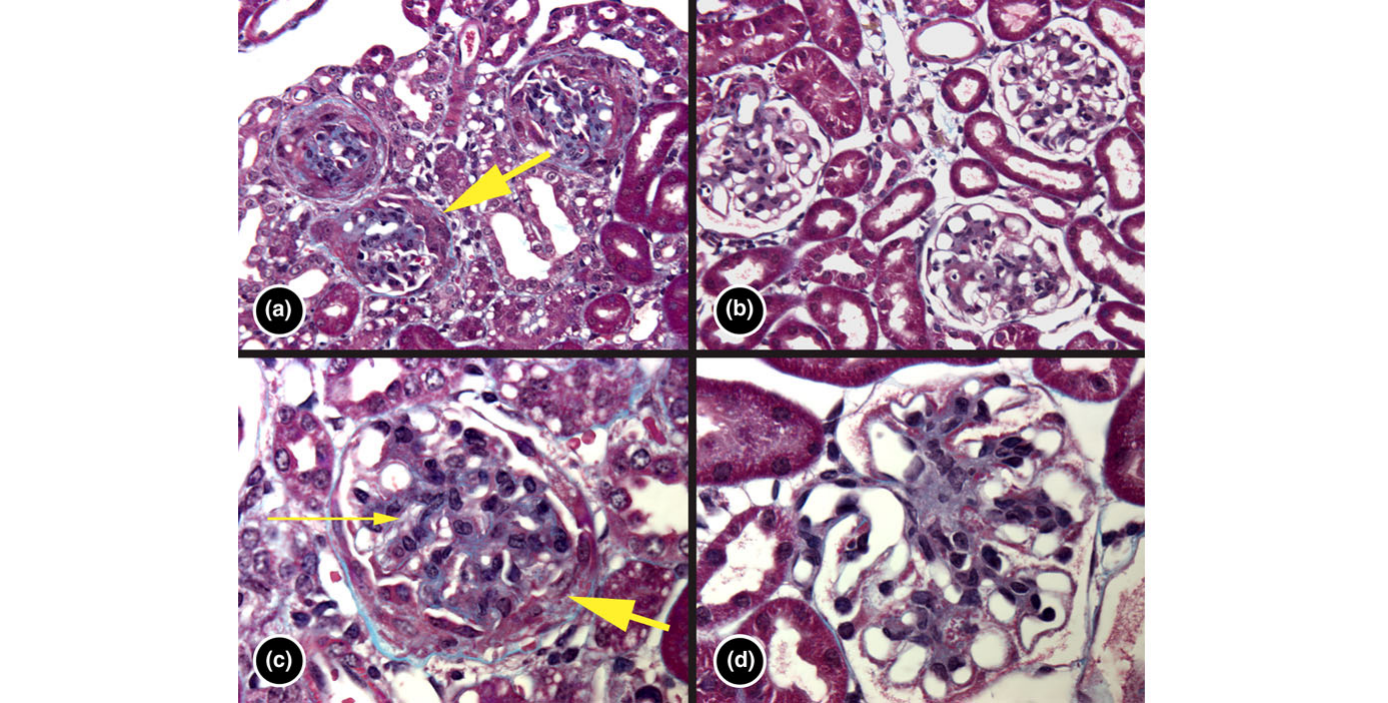
The effect of spironolactone on renal histopathology. Representative light photomicrographs of Masson trichome stained sections from kidneys of **(a,c) **control and **(b,d) **50 mg/kg per day spironolactone treated mice are shown. All of the mice exhibited evidence of proteinaceous, fuchsinophilic deposits in the subepithelial space with thickening of the capillary loops. The mesangium was also expanded in both groups but the cellularity was more commonly increased in the control animals (thin yellow arrow). In addition, the control mice had an active glomerulonephritis with prominent proliferation of extraglomerular cells forming crescents that have begun to organize (thick yellow arrows). Subendothelial deposits and hyaline thrombi were also much more commonly encountered in the control animals. Original magnification: 420× for panels a and b, and 840× for panels c and d.

On histopathologic analysis of hematoxylin and eosin stained and trichrome stained kidney sections, there were marked differences between treated and control mouse kidneys, most pronounced by 36 weeks of age (Figure 3). The kidneys of control mice at 36 weeks exhibited typical features of severe glomerulonephritis, with enlarged glomeruli, mesangial expansion, hypercellularity and deposits, thickened capillary loops with subepithelial deposits, and crescents (Figure 3a,c). The kidneys of mice treated with 50 mg/kg per day spironolactone exhibited less severe glomerulonephritis, with no crescents observed as well as diminished overall cellularity, whereas the capillary loops and mesangium contained less prominent deposits (Figure 3b,d).

**Figure 4:**
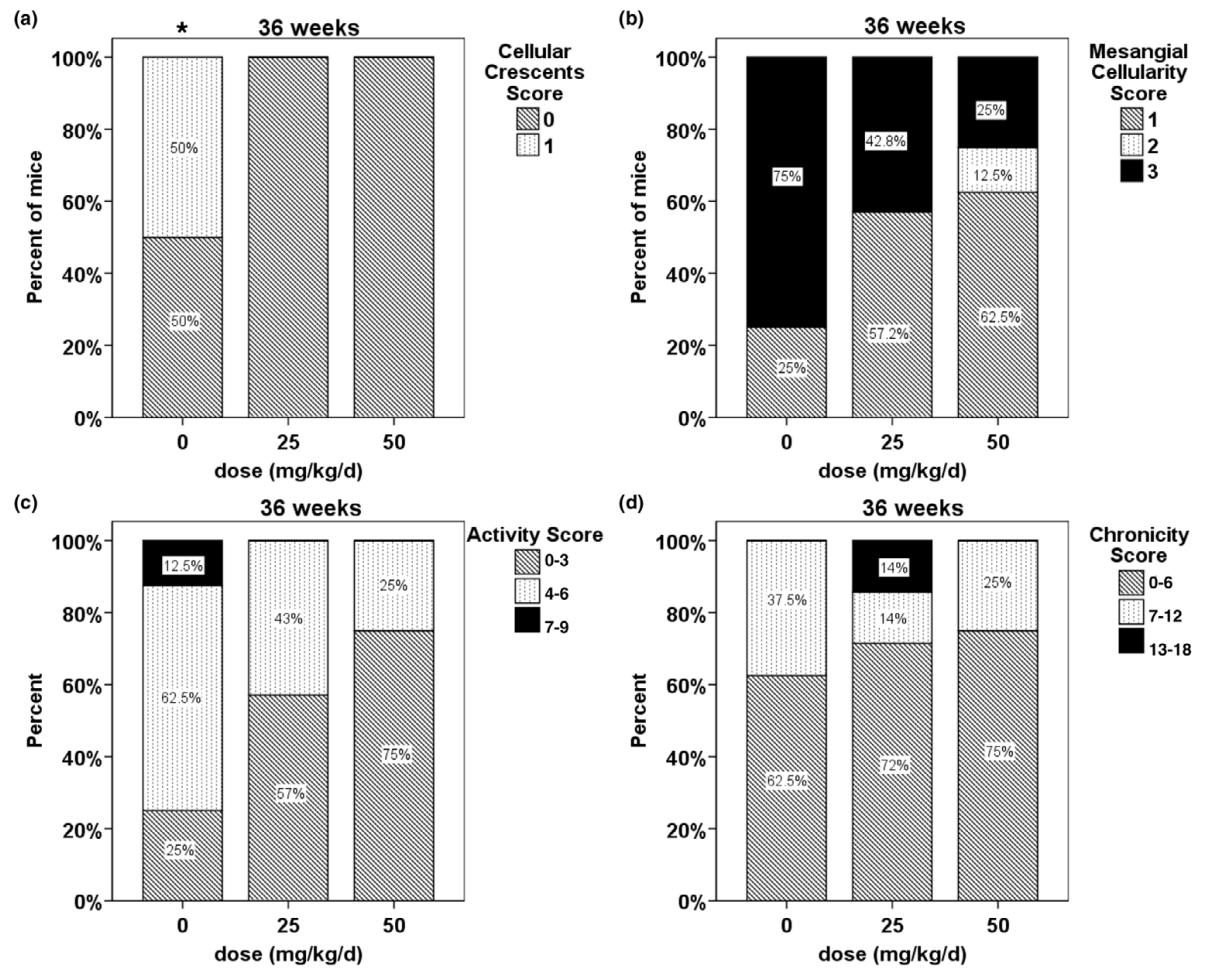
Semiquantitative scoring of histological spironolactone effects. Thirteen renal histopathological parameters were blindly scored from 0 (unaffected) to 3 (severely affected): **(a) **cellular crescent score, **(b) **mesangial cellularity score, **(c) **activity score, and **(d) **chronicity score. At 36 weeks, there were significant differences in cellular crescent formation between treated and control mice (panel a; *P = 0.05) and better scores in treated mice in most other parameters (representative in panel b). A combined score revealed treated mice to have a better activity index (panel c; *P *= 0.10). Chronicity indices were similar compared with control mice (panel D). Data are presented as the percentage of mice in each treatment group that had the indicated score; each treatment group included seven or eight mice by the 36-week time point.

To quantitate histologic differences, a semiquantitative scoring system from 0 to 3 was used to grade 13 different histologic parameters of activity and chronicity. Half of the control mice had cellular crescents present by 36 weeks, whereas none of the treated mice had any (Figure 4a; *P *= 0.05). In most of the other individual parameters assessed (Figure 4b, representative) there was also less prominent disease in treated versus control mice. To assess for relative differences more globally, individual parameters were combined to generate activity (mesangial hypercellularity, mesangial deposits, and endocapillary cellular infiltrate) and chronicity (interstitial fibrosis, tubular atrophy, synechiae, organized crescents, and capillary sclerosis) scores. Again, less histopathologic global activity was observed in treated versus control mice (Figure 4c; *P *= 0.10), with less prominent differences in chronicity scores between treatment groups (Figure 4d).

To determine whether spironolactone treatment resulted in obvious differences in glomerular IC deposition, indirect immunofluorescence staining for IgG and C3 was performed and scored in a blinded manner at 25 weeks of age, which is in the age range when initial florid immune complex deposition occurs [29]. No differences were detected between treated and control mice, despite intense staining for both IgG and C3 in two-thirds of all animals. Additionally, immunohistochemical staining for F4/80 to evaluate for macrophage infiltration was performed, and no significant differences between treatment groups were observed (data not shown). We did not evaluate IC deposition at 36 weeks, because – as we previously reported [27] – it is difficult to detect significant differences in the amount of renal ICs at this later course of the disease, when end-stage has occurred in lupus-prone mice and maximum IC deposition has taken place.

### Gene expression changes with spironolactone

RNA harvested from whole renal tissue was used to perform targeted microarrays, evaluating changes in the expression of approximately 150 cytokine and extracellular matrix-related genes. We found that B lymphocyte stimulator (BLyS) was readily detectable in NZB/W kidneys at age 11 weeks, well before clinical disease manifestations were apparent. Surprisingly, we found no differences in the expression of transforming growth factor-β or other fibrosis-associated genes in our microarray screen at any of our examined time points. We also did not find any major alterations in IFN-α family genes as a result of spironolactone treatment. However, we did find differential expression of a number of genes between treated and control mice, and, based on these semiquantitative results, real-time reverse transcription PCR was performed on selected genes that exhibited consistent changes in expression by spironolactone by microarray analysis and were of potential pathogenic interest. By 36 weeks of age, spironolactone treatment resulted in decreased renal expression (by real-time reverse transcription PCR) of several inflammatory and proapoptotic genes, which was most marked in the 25 mg/kg per day treatment group. Specifically, the fold changes in expression detected in the 25 mg/kg per day treatment group relative to age-matched control mice were as follows: 0.65 for IFN-γ; 0.52 for BlyS; 0.58 for tumor necrosis factor-like weak inducer of apoptosis (TWEAK); 0.70 for tumor necrosis factor related apoptosis-inducing ligand (TRAIL); and 0.49 for Fas ligand.

## Discussion

Treatment of lupus nephritis utilizes immunosuppressives that predominantly target cell-mediated immunity. Because of their potency and nonspecificity, these agents have a variety of undesirable side effects. Additionally, treatment with cytotoxic agents alone may not adequately ameliorate other processes that are involved in progression of kidney disease, which is the primary cause of morbidity/mortality in lupus glomerulonephritis. Thus, adjunctive medication regimens must be developed. Aldosterone is an attractive therapeutic target, given our current understanding of the pathophysiology of lupus nephritis and the clinical utilization of aldosterone blockade in other diseases. We undertook this study to characterize better the effects of aldosterone receptor blockade on different components of progressive murine lupus glomerulonephritis, as proof of concept prior to analyzing ultimately its utility as an adjunctive therapy.

Utilizing the NZB/W F_1 _model, which closely resembles human SLE [29], we found no differences in blood pressure between spironolactone-treated and control mice, which suggests that effects from systemic hypoperfusion were unlikely. Spironolactone did not accelerate the progression of uremia, as indicated by serum blood urea nitrogen levels, which suggests that local renal hemodynamic effects were not detrimental. Hyperkalemia is a well documented consequence of aldosterone blockade, especially in renal insufficiency, and can be a cause of morbidity/mortality [30]. However, serum potassium levels were similar in treated and control mice, despite progressive glomerulonephritis and worsening renal function. Thus, spironolactone could safely be administered on a chronic basis in a model of progressive glomerulonephritis and renal impairment.

The presence of nephrotic range proteinuria indicates significant, although not necessarily irreversible, glomerular damage. In this proof of concept study, we observed a modest but consistently diminished incidence of nephrotic range proteinuria in spironolactone-treated mice. By the final 36-week time point, the treatment groups appeared to be diverging. Prolongation of the study beyond 36 weeks may potentially have resulted in a sustained increase in the incidence of proteinuria in control compared with treated mice. It is also feasible, however, that if spironolactone exerts most of its effects early in the development of glomerulonephritis, then no further diminution in proteinuria onset would be observed at later time points; follow-up studies are needed to address this.

DNA autoAbs, especially to dsDNA, are detected in a subset of patients with SLE, are highly lupus specific, and are pathogenic in lupus nephritis [31-33]. By 36 weeks of age, mice in the 25 mg/kg per day treatment group had significantly lower serum levels of both anti-ssDNA and anti-dsDNA antibodies compared with control mice. However, we did not detect differences in glomerular IC deposition. Therefore, spironolactone does appear to diminish serum levels of anti-DNA antibodies, but this does not result in diminished renal ICs. Potentially, deposition or *in situ *formation of ICs to other, non-DNA, antigens persists and is unaffected by spironolactone treatment. Alternatively, the degree of autoAb diminution may be insufficient to result in clinically detectable decreases in renal ICs. We observed statistically significant differences in serum autoAb levels only at 36 weeks of age, which is after renal IC deposition occurs; thus, it is possible that spironolactone may exert effects on autoAbs that are temporally irrelevant.

The most compelling changes found were in renal histology. Treated mice consistently exhibited less severe histologic evidence of glomerulonephritis compared with control mice. The parameters that exhibited the most improvement were markers of activity (mesangial cellular infiltrates, subepithelial deposits, among others). Although only the presence of cellular crescents was significantly different between treated and control kidneys, scoring of other parameters was highly suggestive of a similar trend. There were less marked differences between treated and control mice in the presence of chronic lesions (tubular atrophy and fibrosis, capillary sclerosis). This was surprising, given that aldosterone receptor blockade is classically thought to ameliorate nonspecific fibrosis and remodeling processes. Potentially, this could relate to the relative robustness of our mice; evaluation at a later time point might have allowed us to detect more chronic changes. Regardless, spironolactone treatment appeared to result in less active (as opposed to chronic) glomerulonephritis. Again, this was observed despite the lack of difference in renal IC deposition. However, other studies in NZB/W mice [34,35] have demonstrated similar uncoupling between renal IC deposition and histological damage. Thus, spironolactone appears to exert its renoprotective effects downstream from the initial insult of IC deposition.

To screen for potential mechanisms by which spironolactone modulates renal damage, we studied RNA expression in kidneys from treated and control mice using targeted microarrays. We found that spironolactone treatment did not have any significant effects on genes associated with fibrosis and extracellular matrix deposition, such as different types of collagens and metalloproteases. With the exception of the genes discussed below, we also did not detect significant differences in renal mRNA expression of other proinflammatory cytokines, despite diminished histopathologic activity in corresponding tissue sections. Potentially, the cytokine signals could have been partially diluted, because we analyzed whole kidney homogenates, not specific regions of the kidney (as can be obtained from laser microdissection.) Thus, lack of spironolactone effect might have been reflective of the low baseline cytokine levels detected.

A few genes potentially relevant to the pathogenesis of lupus nephritis were downregulated by spironolactone treatment. Spironolactone treatment decreased the renal gene expression of IFN-γ and BLyS, which correlated with clinical and histologic findings of diminished SLE glomerulonephritis activity. It has been suggested that the T-helper-1 cytokine IFN-γ plays a critical role in active SLE glomerulonephritis. In humans, intrarenal IFN-γ expression in biopsy specimens from SLE nephritis patients correlated with serologic and histologic activity [36]. Treatment of NZB/W mice with anti-IFN-γ or deletion of the IFN-γ receptor resulted in prolonged survival, diminished frequency and delayed onset of proteinuria, decreased autoAb levels, and decreased histopathologic evidence of glomerulonephritis [37-39]. BLyS, a tumor necrosis factor homolog that stimulates B cell proliferation and maturation, also appears to play a role early in the course of SLE and, potentially, in lupus glomerulonephritis. Higher levels of BLyS are detected in SLE sera and correlate with autoantibody levels [40,41]. NZB/W mice have elevated serum BLyS levels [42], and treatment of NZB/W mice with soluble BLyS receptors results in delayed proteinuria, prolonged survival [42], and decreased serum anti-dsDNA [43]. Thus, the reduction in renal BLyS levels in spironolactone-treated mice could potentially be associated with the downregulation of serum autoantibodies observed by 36 weeks.

We also detected decreases in the expression of the apoptosis-related genes TWEAK, TRAIL, and Fas ligand. This is intriguing, because there is a pathophysiologic basis for a role for aldosterone in apoptosis [44], despite a paucity of studies examining this relationship. TRAIL is upregulated on SLE T cells [45,46] and in lupus serum [47], and has been proposed to mediate tissue damage [46] and nephritis [48]. Urinary TWEAK levels correlate with increased lupus activity [49]. In addition, TWEAK induces mesangial cells to secrete proinflammatory chemokines [50] and induces tubular cell apoptosis in the presence of proinflammatory cytokines [51]; these mechanisms are potentially relevant in the pathogenesis of glomerulonephritis. Fas ligand is also upregulated in the glomeruli [52] and mesangial cells [53] from human lupus glomerulonephritis. All three apoptotic ligands are over-expressed on lupus T cells and induce accelerated APC apoptosis [46]. Thus, diminishment of their levels could result in significant immunomodulation in SLE. This would be a novel mechanism underlying a beneficial effect of spironolactone that must be further explored.

## Conclusion

We describe the effects of aldosterone receptor blockade in a murine model of SLE. Spironolactone is safe and well tolerated, and it appears to improve multiple parameters of lupus activity when it is given early in the course of disease. It may represent a useful therapy in combination with cytotoxic agents, both to improve efficacy and possibly to decrease required treatment dosages. Future studies could expand on the current data by assessing for continued divergence between the treatment groups at later time points, examining the effect of spironolactone on lymphocyte function, performing more localized renal gene array analysis using laser microdissection, and studying the effect of spironolactone in combination with that of cyclophosphamide or mycophenolic acid.

## Abbreviations

autoAb = autoantibody; BlyS = B lymphocyte stimulator; dsDNA = double-stranded DNA; GEO = Gene Expression Omnibus; IC = immune complex; IFN = interferon; PCR = polymerase chain reaction; SLE = systemic lupus erythematosus; ssDNA = single-stranded DNA; TRAIL = tumor necrosis factor related apoptosis-inducing ligand; TWEAK = tumor necrosis factor-like weak inducer of apoptosis.

## Competing interests

The authors declare that they have no competing interests.

## Authors' contributions

SM participated in the study design, performed the majority of the mouse work, immunoassays, renal tissue preparation, microarray analysis, statistical analysis, and drafted the manuscript. PDK performed blinded grading of the histologic specimens. MA performed real-time PCR and analyzed the data. AB assisted with mouse studies and performed the majority of the immunohistochemistry and immunofluorescence work. MJK conceived of the study, participated in its design, data analysis and coordination, and helped to draft the manuscript. All authors read and approved the final manuscript.
